# Book Notices

**Published:** 1894-10

**Authors:** 


### Book Notices.

---

AN ILLUSTATED DICTIONARY OF MEDICINE, BIOLOGY AND ALLIED SCIENCES. Including the Pronunciation, Accentuation, Derivation, and Definition of the Terms Used in Medicine and the Allied Sciences. By George M. Gould, A. M., M. D., Author of "The Student's Medical Dictionary;" "12,000 Medical Words Pronounced and Defined;" "The Meaning and Method of Life," Editor of the Medical News; President, 1893-94, American Academy of Medicine; one of the Ophthalmologists of the Philadelphia Hospital. Philadelphia: P. Blakiston, Son & Co. In one compact, large octavo valume of 1633 pages, bound so as to lie open at any page. Prices, full sheep, net, $10.00; half Morocco, net, $10.00; half Russia, with Thumb Index, net, $12.00.

In calling the attention of our readers to this superb lexicon, we feel that we cannot do better than to reproduce the following from the review in the *University Medical Magazine*, July, 1894:

"The present work is not a revision or a compilation of a previous work, but is entirely new, and is the result of a careful gleaning by the author and corps of assistants of the living literature of the day. The objects which the author has sought to accomplish were, briefly: 1. The inclusion of the many thousands of new words and terms that have been introduced into medicine during the last few years. 2. To give the most complete epitomization of the words of the older and authoritative lexicographers. 3. To include all the more commonly used terms in biology. 4. Keeping the size and purpose of the book well in mind, to give it an encyclopedic character, not only by supplying the usual pronunciation, derivation, and definition of words, but also by showing their logical relations, their bearings, and their practical importance for the worker in literary or clinical medicine. 5. When advisable, to give a pictorial illustration. 6. To cast the influence of the work, in doubtful or disputed cases, in favor of a more consistent and phonetic spelling. 7. To indicate the best pronunciation of words by the simplest and most easily understood method. In scanning through the book with these in mind, we can but say that the work seems to have been well and thoroughly accomplished. The spelling of hemorrhage, orthopedic, etc., with a single "e" instead of with the diphthong will probably seem to some a little radical. It is, however, in line with progress, with the teaching of the leading philologists, and with the most recent and authoritative popular dictionaries. In addition to furnishing concise and accurate definitions and pronunciations, upwards of one hundred tables have been included, in which is classified an immense number of facts collected from various sources. Many of these have been prepared especially for this work, and are not to be found elsewhere. Space will permit us to mention but a few of them. We note a complete table of arteries, giving the origin, distribution and branches of each; a table of bacteria, covering thirty closely-printed pages, and beyond doubt the most complete to be found anywhere; a table showing the expectation of life; a complete table of muscles, giving the origin, insertion, innervation, and function of each; a complete table of nerves, giving the function, origin, distribution and branches; a table of parasites, covering forty-three pages, and, like that of bacteria, the most comprehensive yet collected; a table of tests, embracing thirty-nine pages, giving, beside the name of the test, reagents employed, characteristic reaction, application and remarks; a table of stains and microscopic technique, covering thirty-seven pages, etc. The other tables, while not so large, are correspondingly as complete and valuable. Although the work is so comprehensive, by rigidly economizing space this vast amount of information has been compressed within the limits of one volume of convenient size for handling. We have no hesitation in stating that more useful information is contained in this book than in any other one-volume medical work in the English language—it would probably be safe to say, in any language. While errors are inseparable from human effort, a considerable search failed to disclose any of moment. No argument need here be advanced of the value of a good dictionary. In the one under review, the student of medicine, of dentistry, of veterinary medicine, of biology, of bacteriology, etc., will find an invaluable companion in his reading. From an artistic standpoint, the work is as distinctive as it is in literary merit. The paper is of fine quality, the type and engraving sharp and clear, and the press-work is of a high order. A very valuable feature, and one that will be much appreciated, is that the book opens perfectly flat, and remains open at any point. A useful index refers one to special tables at once, without loss of time."

---

ANTISEPTIC THERAPEUTICS. By Dr. E. L. Trouessart, Paris, France. Translated by E. P. Hurd. "The Physician's Leisure-Library" Series. In two volumes of about 160 pages each. Price per volume, paper, 25 cents; cloth, 50 cents. George S. Davis, Publisher, Detroit, Mich.

This work is based on the microbian theory, and is the result of a demand for an application of antiseptics in general medicine similar to their uses in surgery. It is a matter of some surprise that we should consider asepsis and antisepsis indispensable, and imposed as an absolute rule in general surgical operations, in gynecological practice, in obstetrics, in ophthalmology and rhinology, and should so far neglect these measures in general medicine, even when the disease is known to be of a microbian nature.

There is, beyond question, much to be gained by proper investigation in the line pursued by Dr. Trouessart in these little volumes, and we heartily commend the books to the general profession. H.

---

ESSENTIALS OF PRACTICE OF MEDICINE. By Henry Morris, M. D., with an Appendix on the Clinical and Microscopical Examination of Urine. By Lawrence Wolff, M. D. Colored (Vogel) Urine Scale and numerous fine illustrations. Second edition, enlarged by some three hundred essential formulæ, selected from the writings of the most eminent authorities of the medical profession. Collected and arranged by William M. Powell, M. D. Post 8 vo., 460 pages. Price, cloth, $2.00; medical sheep, $2.50. W. B. Saunders, Publisher, 925 Walnut Street, Philadelphia.

Morris' Practice of Medicine is arranged in the form of questions and answers, an arrangement of especial advantage to the medical student. It contains the essentials of medical practice in a condensed form, and will be found of much benefit to the practicing physician when his time for studying a case is limited. It is carefully prepared, thoroughly reliable, and we can cheerfully recommend it as one of the very best compends on practice. H.

---

INTERNATIONAL CLINICS. A Quarterly of Clinical Lectures on Medicine, Neurology, Pediatrics, Surgery, Genito-Urinary Surgery, Gynecology, Obstetrics, Ophthalmology, Laryngology, Otology and Dermatology, by Professors and Lecturers in the Leading Medical Colleges of the United States, France, Great Britain and Canada. Edited by Judson Daland, M. D., Philadelphia; J. Mitchell Bruce, M. D., F. R. C. P., London, England; David W. Finlay, M. D., F. R. C. P., Aberdeen, Scotland. Volume II, Fourth Series, 1894. Philadelphia. J. B. Lippincott Co. 1894.

The "International Clinics" improves with each volume, and continues to grow in favor with the profession. Volume II of the fourth series contains forty-four lectures by teachers eminent in the medical profession. The following is a partial list of the contributors to this volume: Drs. Henry T. Byford, Henry C. Coe, John B. Hamilton, E. Fletcher Ingalls, George M. Lefferts, G. Frank Lydston, Matthew D. Mann, Charles K. Mills, Paul F. Mundé, Roswell Park, T. Pickering Pick, Thomas R. Pooley,

Prof. Potain, John B. Roberts, A. W. Mayo Robson, B. Sachs, Robert Saundby, Reginald H. Sayre, John C. Shaw, Alexander J. C. Skene, Solomon Solis-Cohen, M. Allen Starr, and a number of others well known to the profession of this country.

We consider these short clinical lectures the most interesting and the most impressive method of teaching medical and surgical practice, and a new volume each quarter, giving the latest facts and practice is a most valuable feature of the work.

The book is of a convenient size, and the mechanical work excellent. H.

---

THE PSYCHOLOGICAL BULLETIN, published by the Medico-Legal Society, and under the management of the *Medico-Legal Journal*, will contain for the month of November (prox.):

"The Study of Sexual Inversion." By Dr. Havelock Ellis, London, England.

"Medical Witnesses. From a Physician's Standpoint." By Dr. Hubbard W. Mitchell, President Medico-Legal Society.

"Hypnotism and Music." Abstracts from a paper, by E. A. Bostwick, in Literary Digest, by the editor.

"Medical Jurisprudence of Insanity; Insanity and Spiritualism," by the editor; and other valuable articles in that line. Those interested in psychological research, should subscribe for the *Bulletin*, it is only $1.50 a year.

---

AN INTRA-MURAL VIEW, a very artistic brochure, has been received from The Curtis Publishing Company, Philadelphia, publishers of *The Ladies' Home Journal*. As the title indicates, the booklet gives us glimpses of the interiors of the *Journal's* offices, and some idea of the work carried on there. The main building, entirely occupied by the editorial and business offices, was designed by Mr. Hardenbergh, the architect of the Hotel Waldorf, New York, and was completed in January, 1893. The exterior is attractive and the interior elegantly appointed and admirably planned. The numerous illustrations, showing the commodious and well-fitted offices, and the accompanying text, giving us some insight into the work of the different bureaus, requiring a force approximating four hundred employees, indicating the wonderful success which *The Ladies' Home Journal* has achieved in an almost incredible short time. The first number was issued in December, 1883, so that less than eleven years have elapsed since Mr. Curtis conceived the idea which has developed into so vast an enterprise. In this short time its merit and steady improvement in all departments have received such recognition that its circulation has reached the enormous average of about 700,000, the largest magazine output in the world. The brochure also describes at some length the work of printing and binding the *Journal*, which is carried on in a separate building. "An Intra-Mural View" will be sent to any one who will address The Curtis Publishing Company and inclose four cents in stamps for postage.

